# Right Ventricle to Pulmonary Artery Coupling Predicts the Risk Stratification in Patients With Systemic Sclerosis-Associated Pulmonary Arterial Hypertension

**DOI:** 10.3389/fcvm.2022.872795

**Published:** 2022-05-11

**Authors:** Jinzhi Lai, Jiuliang Zhao, Kaiwen Li, Xiaohan Qin, Hui Wang, Zhuang Tian, Qian Wang, Mengtao Li, Xiaoxiao Guo, Yongtai Liu, Xiaofeng Zeng

**Affiliations:** ^1^Department of Cardiology, Peking Union Medical College Hospital, Peking Union Medical College and Chinese Academy of Medical Sciences, Beijing, China; ^2^Department of Rheumatology, Peking Union Medical College Hospital, Peking Union Medical College and Chinese Academy of Medical Sciences, Beijing, China

**Keywords:** pulmonary arterial hypertension, systemic sclerosis, right ventricle to pulmonary artery coupling, TAPSE/PASP, echocardiography

## Abstract

**Background:**

Pulmonary arterial hypertension (PAH) is a serious complication of systemic sclerosis (SSc). PAH has high mortality, and risk assessment is critical for proper management. Whether the right ventricle to pulmonary artery (RV-PA) coupling accurately assesses risk status and predicts prognosis in patients with SSc-associated PAH has not been investigated.

**Methods:**

Between March 2010 and July 2018, 60 consecutive patients with SSc-associated PAH diagnosed by right heart catheterization were enrolled prospectively, and the mean follow-up period was 52.9 ± 27.0 months. The RV-PA coupling was assessed by the ratio of tricuspid annular plane systolic excursion (TAPSE) and pulmonary artery systolic pressure (PASP) which was obtained by transthoracic echocardiography. The simplified risk stratification strategy was applied to assess the risk level of participants, and the endpoint was a composite of all-cause death and clinical worsening.

**Results:**

The receiver operating characteristic (ROC) curve of the ability to determine high-risk patients identified the optimal cut-off value of the TAPSE/PASP ratio as 0.194 mm/mmHg, and the ratio appeared to be a reliable indicator in the stratification of patients with high risk (area under the curve = 0.878, ROC *P*-value = 0.003), which showed the highest positive likelihood ratio (LR) (5.4) and the lowest negative LR (0) among a series of echocardiographic parameters. The TAPSE/PASP ratio was an independent predictive factor (HR = 0.01, 95% CI: 0.00–0.77, *P* = 0.037) for the composite endpoint, and patients with a TAPSE/PASP ratio >0.194 had a better overall survival for both the composite endpoint (log-rank χ^2^ = 5.961, *P* = 0.015) and all-cause mortality (log-rank χ^2^ = 8.004, *P* = 0.005) compared to the patients with a TAPSE/PASP ≤ 0.194.

**Conclusion:**

RV-PA coupling assessed by the TAPSE/PASP ratio provides added value as a straightforward and non-invasive approach for predicting risk stratification of patients with SSc-associated PAH. Meanwhile, a lower TAPSE/PASP ratio identified a subgroup with worse prognosis.

## Introduction

Systemic sclerosis (SSc) is a chronic and multisystemic autoimmune disease characterized by vasculopathy, fibrosis, and activation of the immune system, with significant clinical heterogeneity (1). Pulmonary arterial hypertension (PAH) is a common complication of connective tissue disease (CTD). Approximately 74% of CTD-PAH cases are associated with SSc (2), and the prevalence of PAH is approximately 8–12% in SSc, which is much higher than that in other types of CTDs (3). PAH is a leading cause of death in patients with SSc, and a recent study showed that the 1-, 3-, and 5-year survival estimates for these patients were 95, 75, and 63%, respectively (3).

Most SSc-associated PAH patients are diagnosed at an advanced stage, an average of 2 years from symptom onset (1); thus, early diagnosis and identification of disease severity is critically meaningful. Risk assessment plays a key role in disease severity evaluation, prognosis prediction, and treatment guidance for PAH (4). The risk stratification strategy recommended by the 2015 European Society of Cardiology (ESC)/European Respiratory Society (ERS) guidelines is widely used based on a series of multidimensional clinical features (5), while a newly simplified strategy has been proposed recently (6). However, despite its clinical practicability and significance for risk stratification of PAH, invasive right heart catheterization (RHC) is still required to obtain hemodynamic variables.

Recently, the right ventricle to pulmonary artery (RV-PA) coupling assessed by the ratio of tricuspid annular plane systolic excursion (TAPSE) and pulmonary artery systolic pressure (PASP), which was measured by echocardiography has been suggested to be an important clinical and prognostic parameter in patients with heart failure with or without pulmonary hypertension (PH) (7). However, whether this non-invasive and straightforward echocardiographic indicator is able to identify risk stratification in patients with SSc-associated PAH remains uncertain.

## Materials and Methods

### Study Design

Patients with SSc and suspected PH who were referred to our hospital for further screening between March 2010 and July 2018 were included. SSc was confirmed and diagnosed by at least two experienced rheumatologists according to the standard American College of Rheumatology criteria (8) and the contemporary diagnostic criteria for PAH (9), including mean pulmonary arterial pressure (mPAP) ≥25 mmHg at rest, with a pulmonary capillary wedge pressure (PCWP) ≤15 mmHg and pulmonary vascular resistance (PVR) >3 Wood units by RHC. At the 6th World Symposium on Pulmonary Hypertension, in 2018, a working group proposed revising the hemodynamic definition of PH and lowering the threshold of mPAP from ≥25 mmHg to >20 mmHg (10). Since our cohort was recruited mostly before 2018, we used the previous criteria for PAH. Comprehensive transthoracic echocardiography was performed 2 h prior to the RHC.

The exclusion criteria included:

1. Significant valvular heart disease confirmed by echocardiography (moderate to severe stenosis or insufficiency)
2. Congenital heart disease
3. Left ventricular systolic dysfunction, defined as a left ventricle (LV) ejection fraction <50% from the biplane Simpson method by echocardiography
4. Overlap syndrome or antiphospholipid syndrome
5. Suspected chronic thromboembolic disease on computed tomography scans or radionuclide ventilation and perfusion imaging
6. Patients with any other diseases known to be associated with precapillary PH, including PH due to severe respiratory diseases, interstitial lung disease (ILD), chronic thromboembolic PH, or other miscellaneous causes of PAH.

Interstitial lung disease-associated PH was defined if patients had a total lung capacity (TLC) <60% predicted or a TLC between 60 and 70% predicted, combined with moderate to severe fibrosis on high-resolution computed tomography (HRCT) in the absence of pulmonary infections, cardiac diseases, and drug-related changes (11).

The study was approved by the Ethics Committee of the Peking Union Medical College Hospital (No. S-191), conformed to the principles of the Declaration of Helsinki, and followed the International Conference on Harmonization Guidelines for Good Clinical Practice. Written informed consent was obtained from all included patients.

Baseline assessment of eligible SSc-associated PAH patients included clinical parameters [disease duration, ILD, Raynaud phenomenon, gastroesophageal reflux disease (GERD), hypersensitive C-reactive protein (hsCRP), erythrocyte sedimentation rate (ESR), TLC, forced vital capacity (FVC), dispersion of lung carbon monoxide (DLCO) of pulmonary function], transthoracic echocardiography, and RHC. Disease duration was defined as the period from onset of the first non-Raynaud phenomenon syndrome to the diagnosis of PAH.

The study endpoint was a composite of all-cause mortality or hospitalization for worsening PAH (including worsening congestive heart failure, syncope, lung transplantation, or the initiation of parenteral prostacyclin analog therapy) (12).

### Risk Stratification Assessment

The risk analysis of PAH was based on an abbreviated version of the 2015 ESC/ERS risk stratification strategy according to the 6th Symposium on Pulmonary Hypertension (4, 13). Four criteria were used: I, World Health Organization (WHO) functional classification; II, 6-min walk distance (6MWD); III, the level of N-terminal pro B-type natriuretic peptide (NT-proBNP)/B-type natriuretic peptide (BNP) or right arterial pressure (RAP); and IV, cardiac index (CI) or venous oxygen saturation (SvO2). “Low-risk” was defined as meeting at least three low risk criteria and no high-risk criteria; “intermediate-risk” was defined as not belonging to low nor high risk; and “high-risk” was defined as meeting two high risk criteria, including CI or SvO2 (Supplementary Material).

### Hemodynamic Evaluation

Right heart catheterization was performed via an 8.5-Fr introducer sheet in the internal jugular vein or the left subclavian vein and an advanced 6-lumen 8-Fr Swan-Ganz catheter (Edwards Lifesciences World Trade, Irvine, CA, United States) in the pulmonary artery, to measure RAP, mPAP, and PCWP. Cardiac output (CO) was evaluated using the thermodilution technique (Edwards Lifesciences World Trade), at least in triplicate, with a variation of <10% between the measured values. PVR was calculated as follows: PVR = (mPAP-PCWP)/CO. Mixed venous blood was obtained from the pulmonary arteries during RHC.

### Echocardiography

Baseline transthoracic echocardiography was performed with an ultrasound system (Vivid 7 or E9, GE Vingmed Ultrasound, Horten, Norway), and echocardiographic recordings were obtained with a 1.6–3.2 MHz transducer following the guidelines of the American Society of Echocardiography (14). Offline analysis was conducted using off-line dedicated software (EchoPAC, GE Vingmed Ultrasound).

The structure and function of the right ventricle (RV) were evaluated according to a report from the American Society of Echocardiography (15), including the measurement of the right ventricular transverse diameter (RVTd), right ventricular length diameter (RVLd), right atrial (RA) area, TAPSE, right ventricular fractional area change (RVFAC), tricuspid valve lateral annular peak systolic velocity (TVS’), and RV Tei index. TAPSE was measured at the lateral tricuspid annulus using M-mode imaging in the RV-focused apical four-chamber view. PASP was calculated using the following formula: pressure gradient derived from peak tricuspid regurgitant velocity + estimated RA pressure. RVTd and RVLd were measured from the apical four-chamber view at end-diastole, and the RA area was acquired at end-systole. Right ventricle end-diastolic and end-systolic areas were measured from the same view at end-diastole and end-systole, respectively. RVFAC was derived as follows: RVFAC = [(right ventricle end-diastolic area − right ventricle end-systolic area) / right ventricle end-diastolic area] × 100%. Pulse-wave tissue Doppler imaging (TDI) was performed to obtain tricuspid annular velocities by setting the same point on the lateral side of the tricuspid annulus. The measurements included TVS’. The RV Tei index was calculated using the following formula: [(isovolumic contraction time + isovolumic relaxation time) / ejection time]. The TAPSE/PASP ratio was calculated to indicate the RV-PA coupling.

Myocardial strain was evaluated on a frame-by-frame basis by automatic tracking of acoustic markers throughout the cardiac cycle. The peak systolic strain was defined as the maximum value of the peak negative strain (myocardial shortening) during systole. RV longitudinal strain (RVLS) was assessed in the RV-focused apical four-chamber view and was acquired by averaging three segments of the RV free wall. Two-dimensional strain parameters were evaluated in both the subendocardial and subepicardial layers of the myocardium.

All echocardiographic recordings and measurements were performed by two experienced cardiologists who were blinded to the clinical diagnosis and endpoints.

### Treatment and Clinical Follow-Up

Pulmonary arterial hypertension-targeted therapy, including treatment with an endothelin receptor antagonist, phosphodiesterase type 5 inhibitor, and prostacyclin analog, was initiated with appropriate consideration by experienced rheumatologists according to recommendations (9). Glucocorticoids and immunosuppressants were administered when required for underlying SSc. Patients were followed up in our center every 3–6 months after hospital discharge. At the end of this study (30 November 2019), the status of each patient was confirmed by a review of their medical records and a telephone visit. Patients were excluded if they dropped out during follow-up.

The study endpoint was a composite of all-cause death and hospitalization for worsening PAH (including worsening congestive heart failure, syncope, lung transplantation, or the initiation of parenteral prostacyclin analog therapy). The survival time was calculated as the time from baseline echocardiography to the end of the study (30 November 2019) or the composite endpoint.

### Data Analysis

Continuous variables are expressed as mean ± standard deviation (SD), and categorical variables are expressed as frequency (percentage). Non-normally distributed data are expressed as the median [interquartile range (IQR)] using the Kolmogorov–Smirnov test.

Receiver-operating characteristic (ROC) analysis, area under the curve (AUC), sensitivity, specificity, positive predictive value (PPV), negative predictive value (NPV) and likelihood ratio (LR) were used to determine the diagnostic accuracy of risk stratification from significant echocardiographic variables in patients with SSc-associated PAH. The optimal cut-off value of TAPSE/PASP was calculated, and all subjects were divided into two groups according to the optimal cut-off (≤0.194 and >0.194 mm/mmHg). Baseline information, including demographic and clinical characteristics, hemodynamic parameters, echocardiographic variables, and endpoint events, was compared between the two groups using the two-tailed *t*-test or Mann–Whitney U test, and proportions were compared using the Chi-square test or Fisher’s exact test. Clinical parameters and the ratio of TAPSE/PASP with the prognostic composite endpoint was evaluated with a Cox proportional hazards regression model for univariate analysis, and the variables with *P* ≤ 0.05 in the univariate analysis were selected for the multivariate analysis. Overall survival and endpoint event-free rates were further assessed by Kaplan–Meier analysis with comparisons performed by the log-rank test to validate the prediction efficiency of the TAPSE/PASP ratio.

Statistical significance was considered at a two-tailed *P*-value < 0.05, and all analyses were performed using SPSS software (version 21.0, SPSS, Inc., IBM).

## Results

### Patient Characteristics

**FIGURE 1:**
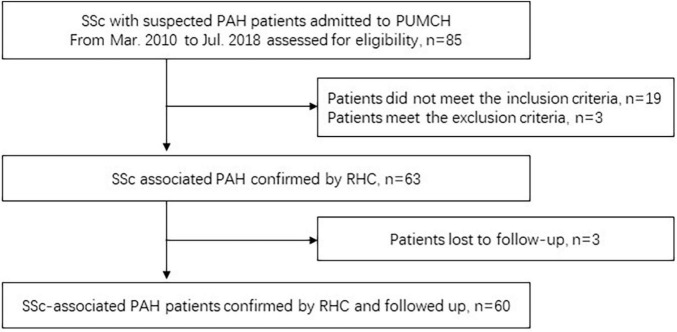
Flowchart of patient screening. SSc, systemic sclerosis; PUMCH, Peking Union Medical College Hospital; RHC, right heart catheterization.

A total of 85 patients were screened, and 60 consecutive patients were finally enrolled and analyzed, same as our previous study (BR16a), and shown in Figure 1. The baseline clinical characteristics and outcomes are described in Table 1. Most of the patients were female (91.7%) and the mean age at PAH diagnosis was 44.7 ± 11.3 years. Among these patients, 87.9% presented with Raynaud phenomenon, 76.8% had ILD, and 46.4% had GERD. The mPAP was 38.0 (IQR: 32.0, 49.8) mmHg, and the CO was 3.9 (IQR: 3.6–5.2) L/min. According to the abbreviated version of the 2015 ESC/ERS risk stratification strategy, 37 (61.7%) belonged to the low-risk group, 17 (28.3%) patients were classified as intermediate-risk, and 6 (10.0%) were in the high-risk group.

**TABLE 1:** Baseline characteristics and comparison of variables based on optimal cut-off for RV-PA coupling.

	Total (*n* = 60)	TAPSE/PASP ≤ 0.194 (*n* = 17)	TAPSE/PASP > 0.194 (*n* = 43)	*P*-value
**Demographic and clinical variables**				
Age at PAH diagnosis (years)	44.7 ± 11.3	44.7 ± 11.4	44.6 ± 11.4	0.981
Disease duration (months)	24.5 (10.0–84.0)	20.5 (6.8–66.0)	36.00 (12.3–84.0)	0.109
Women, *n* (%)	55 (91.7)	16 (94.1)	39 (90.7)	0.666
BMI (kg/m^2^)	19.8 (18.0–22.5)	20.1 (19.0–22.0)	19.5 (18.0–22.7)	0.541
Disease subset (lcSSc) (%)	39/52 (75.0)	12/13 (92.3)	27/39 (69.2)	0.096
mRSS	2.0 (0.0–7.8)	0.0 (0.0–3.0)	2.0 (0.0–11.0)	0.500
Digital ulcers (%)	11/45 (24.4)	3/14 (21.4)	8/31 (25.8)	0.752
Pitting scars (%)	9/45 (20.0)	3/14 (21.4)	6/31 (19.4)	0.872
ILD, *n* (%)	43/56 (76.8)	11/16 (68.8)	32/40 (80.0)	0.368
Raynaud phenomenon, *n* (%)	51/58 (87.9)	17 (100.0)	34/41 (82.9)	0.069
GERD, *n* (%)	26/56 (46.4)	7/16 (43.8)	19/40 (47.5)	0.799
PAH–targeted therapy, *n* (%)	47/58 (81.0)	15 (88.2)	32/41 (78.1)	0.368
ESR (mm/h)	23.0 (7.0–38.0)	14.5 (5.0–26.0)	23.0 (8.0–40.0)	0.102
hsCRP (mg/L)	1.8 (0.7–6.1)	2.1 (1.1–7.4)	1.8 (0.5–6.3)	0.585
GFR (ml/min)	108.1 (99.5–117.5)	101.4 (90.1–112.2)	108.6 (101.5–117.8)	0.185
WHO-FC III/IV (%)	24/58 (41.4)	9/16 (56.3)	15/42 (35.7)	0.156
TLC predicted (%)	85.4 (73.9–91.6)	85.4 (80.0–94.8)	85.0 (67.2–91.7)	0.503
FVC predicted (%)	80.0 (67.0–86.0)	82.6 (68.9–90.9)	79.3 (64.9–85.0)	0.252
DLCO predicted (%)	50.3 ± 15.0	49.84 ± 7.03	50.48 ± 17.08	0.896
**Hemodynamic variables**				
mPAP (mmHg)	38.0 (32.0–49.8)	51.0 (43.0–56.0)	34.0 (30.0–41.0)	<0.001
PCWP (mmHg)	8.0 (6.0–10.0)	9.0 (7.0–10.0)	7.0 (4.8–10.0)	0.041
CO (L/min)	3.9 (3.6–5.2)	3.6 (3.2–3.8)	4.3 (3.7–5.5)	0.002
PVR (Wood units)	7.5 (4.9–11.1)	11.9 (9.2–14.5)	6.7 (4.7–8.6)	<0.001
**Echocardiographic parameters**				
RVTd (mm)	40.1 ± 6.1	45.9 ± 5.0	38.3 ± 5.1	<0.001
RVLd (mm)	63.8 ± 10.0	66.7 ± 8.0	63.0 ± 10.5	0.221
TVS’ (cm/s)	10.5 ± 2.4	8.4 ± 2.4	11.1 ± 2.1	<0.001
RV Tei index	0.53 ± 0.18	0.56 ± 0.19	0.52 ± 0.17	0.392
RVFAC (%)	34.8 ± 13.4	24.6 ± 11.8	37.9 ± 12.4	0.001
TAPSE (mm)	16.0 ± 3.0	12.4 ± 2.1	17.1 ± 3.2	<0.001
RVLS (free wall) (%)	19.9 ± 7.9	14.0 ± 5.6	21.7 ± 7.7	0.001
PASP (mmHg)	61.2 ± 18.2	84.0 ± 11.3	54.2 ± 13.5	<0.001
**Treatment and endpoint events**				
PAH-targeted therapy, *n* (%)	47/58 (81.0)	17 (88.2)	32/41 (78.1)	0.368
All-cause mortality, *n* (%)	22 (36.7)	11 (64.7)	11 (25.6)	0.005
Composite endpoint, *n* (%)	28 (46.7)	12 (70.6)	16 (37.2)	0.02

*RV-PA coupling, right ventricle to pulmonary artery coupling; BMI, body mass index; CO, cardiac output; DLCO, dispersion of lung carbon monoxide; ESR, erythrocyte sedimentation rate; FVC, forced vital capacity; GERD, gastroesophageal reflux disease; GFR, glomerular filtration rate; hsCRP, hypersensitive C-reactive protein; ILD, interstitial lung disease; lcSSc, limited cutaneous systemic sclerosis; mPAP, mean pulmonary arterial pressure; mRSS, modified Rodnan skin score; NT-proBNP, N-terminal pro B-type natriuretic peptide; PAH, pulmonary arterial hypertension; PASP, pulmonary arterial systolic pressure; PCWP, pulmonary capillary wedge pressure; PVR, pulmonary vascular resistance; RV, right ventricle; RVFAC, right ventricle fractional area change; RVLd, right ventricular length diameter; RVLS, right ventricle longitudinal strain; RVTd, right ventricular transverse diameter; TAPSE, tricuspid annular plane systolic excursion; TLC, total lung capacity; TVS’, tricuspid valve lateral annular peak systolic velocity; WHO-FC, World Health Organization functional classification.*

All patients received immunosuppressive treatment, and 47 (81.0%) patients were administered PAH-targeted medications. During a mean follow-up period of 52.9 ± 27.0 months, 22 patients (36.67%) experienced all-cause death after 32.1 ± 19.8 months and 28 (46.67%) experienced a composite endpoint after 23.0 (IQR: 9.0–54.5) months.

### Patients With Different Tricuspid Annular Plane Systolic Excursion/Pulmonary Artery Systolic Pressure Ratios

**FIGURE 2:**
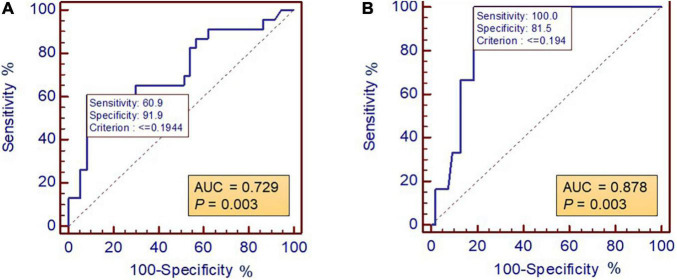
Receiver operating characteristic curve showed the optimal cut-off for RV-PA coupling to determine SSc-PAH patients with stratification of **(A)** intermediate and high risk and **(B)** high risk. ROC, receiver operating characteristic; RV-PA, right ventricle to pulmonary artery.

The ROC curve for the ability to determine high- and low-risk patients identified the optimal cut-off for the TAPSE/PASP ratio as 0.194 mm/mmHg, as shown in Figure 2. The baseline characteristics of the study cohort, grouped according to the cut-off value of the TAPSE/PASP ratio are shown in Table 1. There were no significant differences in the demographic and clinical variables between the two groups. However, in the group with a TAPSE/PASP ≤ 0.194, the levels of mPAP (*P* < 0.001), PCWP (*P* = 0.041), and PVR (*P* < 0.001) were significantly higher than those in the group with TAPSE/PASP > 0.194, while the level of CO (*P* = 0.002) was significantly lower in the group with TAPSE/PASP ≤ 0.194. As for the echocardiographic parameters, patients with a TAPSE/PASP > 0.194 had significantly higher levels of TVS’ (*P* < 0.001), RVFAC (*P* = 0.001), TAPSE (*P* < 0.001), and RVLS (*P* = 0.001) than those with a TAPSE/PASP ≤ 0.194. Meanwhile, the patients in the smaller TAPSE/PASP ratio group showed a higher proportion of all-cause death (64.7 vs. 25.6%, *P* = 0.005) or a composite endpoint (70.6 vs.37.2%, *P* = 0.02).

### Tricuspid Annular Plane Systolic Excursion/Pulmonary Artery Systolic Pressure Ratio and Risk Stratification of Patients With Systemic Sclerosis-Associated Pulmonary Arterial Hypertension

Table 2 shows the area under the ROC curve, sensitivity, specificity, positive and NPVs, and LR + LR-efficiency for the suggested cut-off points for TAPSE/PASP ratio and other echocardiographic parameters to determine the risk stratification of patients with SSc-associated PAH. These echocardiographic parameters describe the performance of the right ventricle and may be related to disease severity. In the stratification of high-risk patients, the TAPSE/PASP ratio emerged as a reliable indicator (AUC = 0.878, ROC *P* = 0.003), and the cut-off value showed the highest positive LR (5.4) and lowest negative LR (0). At the same time, the cut-off value of the TAPSE/PASP ratio was excellent in identifying low-risk patients with the highest positive LR (7.51) and very low negative LR (0.43) (Table 2).

**TABLE 2:** Receiver operating characteristic analysis and diagnostic accuracy of significant echocardiographic parameters in the prediction of risk stratification for patients with SSc-associated PAH.

	AUC	ROC *P*-value	Cut-off point	Sensitivity (%)	Specificity (%)	PPV (%)	NPV (%)	LR +	LR−
	**Diagnosis of intermediate and high risk for patients with SSc-associated PAH**								
RVTd (mm)	0.829	<0.001	>42	65.2	89.2	79	80.5	6.03	0.39
RVLd (mm)	0.747	0.001	>66	78.3	73	64.3	84.4	2.9	0.3
TVS’ (cm/s)	0.682	0.019	≤9	52.2	78.4	60	72.5	2.41	0.61
RV Tei index	0.598	0.204	>0.4095	87	43.2	48.8	84.2	1.53	0.3
RVFAC (%)	0.666	0.031	≤30.4	60.9	75.7	60.9	75.7	2.5	0.52
TAPSE (mm)	0.722	0.004	≤14	60.9	75.7	60.9	75.7	2.5	0.52
PASP (mmHg)	0.643	0.065	>60	60.9	67.6	53.9	73.6	1.88	0.58
TAPSE/PASP (mm/mmHg)	0.729	0.003	≤0.1944	60.9	91.9	82.4	79.1	7.51	0.43
RVLS (free wall) (%)	0.678	0.022	≤19.54	73.9	63.9	56	79.8	2.05	0.41
RVLS (free wall) (%)/PASP (mmHg)	0.685	0.017	≤0.2283	56.5	77.8	61.3	74.2	2.54	0.56
	**Diagnosis of high risk for patients with SSc-associated PAH**								
RVTd (mm)	0.843	0.006	>42	83.3	74.1	26.3	97.6	3.21	0.22
RVLd (mm)	0.827	0.009	>67.4	100	68.5	26.1	100	3.18	0
TVS’ (cm/s)	0.731	0.065	≤9	83.3	72.2	25	97.5	3	0.23
RV Tei index	0.586	0.49	>0.6255	50	79.6	21.4	93.5	2.45	0.63
RVFAC (%)	0.682	0.146	≤30.4	83.3	66.7	21.7	97.3	2.5	0.25
TAPSE (mm)	0.836	0.007	≤13	83.3	79.6	31.2	97.7	4.09	0.21
PASP (mmHg)	0.756	0.072	>63.2	83.3	66.7	21.7	97.3	2.5	0.25
TAPSE/PASP (mm/mmHg)	0.878	0.003	≤0.194	100	81.5	37.5	100	5.4	0
RVLS (free wall) (%)	0.717	0.084	≤18.59	83.3	60.4	18.9	97	2.1	0.28
RVLS (free wall) (%)/PASP (mmHg)	0.745	0.05	≤0.201	66.7	79.2	59.7	83.8	3.21	0.42

*AUC, area under the curve; LR+, positive likelihood ration; LR−, negative likelihood ratio; NPV, negative predictive value; PAH, pulmonary arterial hypertension; PASP, pulmonary arterial systolic pressure; PPV, positive predictive value; ROC, receiver-operating characteristic; RV, right ventricle; RVLd, right ventricular length diameter; RVTd, right ventricular transverse diameter; RVFAC, right ventricle fractional area change; SSc, systemic sclerosis; TAPSE, tricuspid annular plane systolic excursion; TVS’, tricuspid valve lateral annular peak systolic velocity; RVLS, right ventricle longitudinal strain.*

### Predictors for the Composite Endpoint

Among all the clinical variables, WHO-FC III/IV (HR = 2.42, 95% CI: 1.13–5.18, *P* = 0.023), plasma levels of NT-proBNP (HR = 1.02, 95% CI: 1.00–1.04, *P* = 0.026) and the TAPSE/PASP ratio (HR = 0.01, 95% CI: 0.00–0.43, *P* = 0.015) were predictive factors for the composite endpoint (Table 3). In the multivariate Cox proportional hazard analysis, the parameters with *P* ≤ 0.05 in the univariate analysis were selected for the multivariate analysis. After adjusting WHO-FC III/IV and NT-proBNP, the TAPSE/PASP ratio (HR = 0.01, 95% CI: 0.00–0.77, *P* = 0.037) was still an independent predictive factor of the composite endpoint.

**TABLE 3:** Prognostic analysis for the association between TAPSE/PASP and composite endpoint.

Variables	χ^2^	Hazard ratio (95% CI)	*P*-value
**Univariate analysis**			
Gender (female)	2.78	2.49 (0.85–7.25)	0.095
Disease duration	0.06	0.99 (0.99–1.01)	0.800
BMI (kg/m^2^)	0.37	0.97(0.88–1.07)	0.546
ILD	0.50	0.73 (0.30–1.76)	0.480
Raynaud phenomenon	2.92	0.43 (0.16–1.13)	0.087
GERD	1.43	1.62 (0.74–3.56)	0.233
GFR (ml/min)	0.34	0.99 (0.98–1.01)	0.559
NT-proBNP (every 100 ng/L)	4.96	1.02 (1.00–1.04)	0.026
hsCRP (mg/L)	2.23	1.03 (0.99–1.07)	0.135
WHO-FC III/IV	5.18	2.42 (1.13–5.18)	0.023
TLC predicted (%)	1.44	0.99 (0.96–1.01)	0.230
FVC predicted (%)	0.07	1.00 (0.98–1.03)	0.789
DLCO predicted (%)	2.72	0.97 (0.94–1.01)	0.099
TAPSE/PASP (mm/mmHg)	5.93	0.01 (0.00–0.43)	0.015
**Multivariate analysis**			
WHO-FC III/IV	2.84	2.10 (0.89–4.97)	0.092
NT-proBNP (every 100 ng/L)	1.80	1.01 (1.00–1.03)	0.179
TAPSE/PASP	4.34	0.01(0.00–0.77)	0.037

*BMI, body mass index; FVC, forced vital capacity; GERD, gastroesophageal reflux disease; GFR, glomerular filtration rate; hsCRP, hypersensitive C-reactive protein; ILD, Interstitial lung disease; NT-proBNP, N-terminal pro B-type natriuretic peptide; PASP, pulmonary arterial systolic pressure; TAPSE, Tricuspid annular plane systolic excursion; TLC, total lung capacity; WHO-FC, World Health Organization functional classification.*

### Tricuspid Annular Plane Systolic Excursion/Pulmonary Artery Systolic Pressure Ratio and Endpoint-Free Event

**FIGURE 3:**
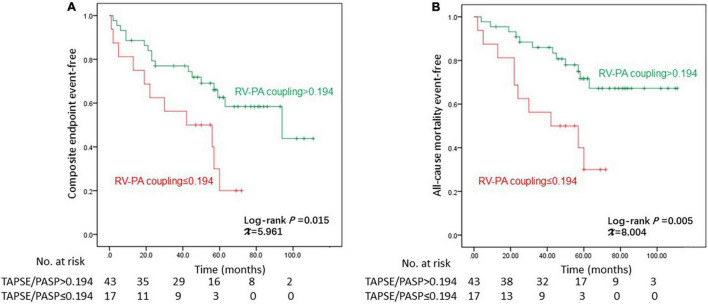
Kaplan–Meier curves for the probability of **(A)** composite endpoint and **(B)** all-cause mortality. PASP, pulmonary artery systolic pressure; TAPSE, tricuspid annular plane systolic excursion; RV-PA, right ventricle to pulmonary artery.

The significance of the TAPSE/PASP ratio in predicting the outcomes of patients with SSc-associated PAH was further confirmed by Kaplan–Meier analysis, as shown in Figure 3. Patients with TAPSE/PASP >0.194 had a better overall survival for both the composite endpoint (log-rank χ^2^ = 5.961, *P* = 0.015) and all-cause mortality (log-rank χ^2^ = 8.004, *P* = 0.005) than those with TAPSE/PASP ≤0.194.

## Discussion

In our prospective cohort, we assessed the feasibility of using echocardiographic parameters to stratify the risk status and indicate the prognosis of patients with SSc-associated PAH. Our results showed that the TAPSE/PASP ratio, which is a non-invasive and easy-to-use index measured by echocardiography, determines the risk stratification of patients with SSc-PAH with reliable diagnostic accuracy, while a low TAPSE/PASP ratio predicts patients with poor prognosis. Here, we provide an alternative index for invasive RHC measurements at the bedside.

Risk assessment plays a vital role in managing patients with PAH. The strong relationship between risk stratification, prognosis, and therapy considerations, including initial strategy and further escalation, has been emphasized and serves as the rationale for a treatment strategy that is based on disease severity as assessed by a multi-parametric risk stratification approach (17). Over time, different baseline and follow-up parameters have been utilized individually or combined in formulas or calculators to predict outcomes. Among these variables, including functional classification, 6MWD, NT-proBNP, and BNP plasma level, the CI, RAP, and SvO2, are the highest yields in the series of registry analyses (4, 13, 18, 19). CI, RAP, and SvO2 are appropriate at both baseline and first follow-up evaluation, and fulfilment of low-risk criteria is associated with a good prognosis (20). Therefore, a new simplified risk stratification strategy based on four criteria and these six variables has recently been proposed (6). We realize that risk stratification is a key point not only for severity assessment but also for the treatment strategy of PAH according to previous studies; however, non-invasive echocardiographic parameters that predict risk stratification are rarely reported. Our study revealed that a smaller TAPSE/PASP ratio (≤0.194 mm/mmHg) provided reliable diagnostic accuracy for identifying high-risk criteria in patients with SSc-associated PAH.

The RV is a major determinant of the functional state and prognosis of PAH. The RV adapts to the increased afterload in PAH by increasing contractility to preserve RV-arterial coupling and flow output response to peripheral demand (21). Some patients develop adaptive RV hypertrophy, while others develop maladaptive RV hypertrophy with dilatation, fibrosis, and RV failure (22). RV-PA coupling refers to the relationship between RV contractility and RV afterload. In the clinical context, RV-PA coupling is most widely measured by arterial elastance (Ea; a measure of afterload) and ventricular elastance (Emax; a measure of contractility), which are determined invasively using pressure-volume loop analysis (23). However, this approach is expensive, technically demanding, and impractical at the bedside. As a result, evaluating the functional state of PAH remains a difficult endeavor given the overall low prevalence in the general population (24). An easier and more practical approach needs to be applied to screening for PAH in the SSc population during diagnosis and follow-up. In a recent study of 52 patients with idiopathic PAH, TAPSE/PASP was proposed to be a clinically relevant and valid surrogate of invasively measured Ees/Ea to assess RV-PA coupling and provide information on RV diastolic stiffness (21). Our previous study showed that the TAPSE/PASP ratio provided relevant prognostic insights in patients with SLE-associated PAH. In particular, a low TAPSE/PASP and low 6MWD identified a subgroup of patients with a high risk of poor prognosis (25). Moreover, the functions of the TAPSE/PASP ratio in assessing the severity of other diseases have been validated, such as in chronic lung disease (26) and the model for end-stage liver disease (MELD) (27). A recent study of 92 patients with coronavirus disease (COVID-19) also showed that bedside echocardiography of TAPSE/PASP adds to the prognostic relevance of PaO2/FiO2 in acute respiratory distress syndrome (ARDS) (28). The cut-off value of TAPSE/PASP ratio in present study is comparable to the ratio in the cohort study which included patients with PAH (0.19 mm/mmHg) (7), and our previous report on patients with SLE-associated PAH (0.184 mm/mmHg) (25). However, the value is much lower than the ratio in patients with left-sided heart failure (0.35 mm/mmHg) (29), chronic lung disease (0.26 mm/mmHg) (26) and ARDS due to COVID-19 (0.635 mm/mmHg) (28). One possible explanation for these findings is that the RV-PA uncoupling in patients with left heart failure, ARDS and chronic lung disease is always involved in severe LV dysfunction or pulmonary parenchyma injury while in PAH population the left heart systolic function and pulmonary function usually preserved.

Taken together, our study revealed evidence-based validation of echocardiographic variables to determine risk status and predict prognosis in patients with SSc-associated PAH and implied that the TAPSE/PASP ratio is potentially appropriate for further clinical applications in severity identification, treatment decision, prognosis prediction, and follow-up.

## Limitations

Our study has several limitations. First, this was a single-center study with a prospective design and limited sample size. Our optimal cut-off values for diagnosing high-risk and low-risk criteria were the same because of the small sample size of high-risk patients. Therefore, the validity of the identified cut-off value of the TAPSE/PASP ratio should be further confirmed by larger multicenter cohorts to provide stronger evidence. Second, TAPSE is a preload-dependent measure that reflects the function of longitudinal motion for RV. As RV dysfunction progresses, TAPSE reaches a minimum and shows no further decrease (30). Thus, the reliability of the TAPSE/PASP ratio may be reduced in the very late stage of RV failure. Third, although our study showed a significant correlation between baseline TAPSE/PASP ratio and all-cause mortality, further application of this parameter in subsequent follow-up should be performed to accumulate clinical experience.

## Conclusion

In conclusion, RV-PA coupling assessed by the echocardiography-derived TAPSE/PASP ratio provided a non-invasive and easy-to-use indicator for determining risk stratification with reliable diagnostic efficiency in patients with SSc-associated PAH. Meanwhile, a lower TAPSE/PASP ratio identified a subgroup with worse prognosis.

## Data Availability Statement

The raw data supporting the conclusions of this article will be made available by the authors, without undue reservation.

## Ethics Statement

The studies involving human participants were reviewed and approved by the Ethics Committee of the Peking Union Medical College Hospital (No. S-191). The patients/participants provided their written informed consent to participate in this study.

## Author Contributions

JL, JZ, XQ, HW, ZT, QW, ML, and XG performed the data collection. JL and JZ performed the statistical analysis. JL performed the result interpretation. YL and XZ performed the conceptualization. JL, KL, and XG wrote the manuscript. XG and YL provided the fund support. All authors contributed to the study conception and design, read and approved the final manuscript.

## Conflict of Interest

The authors declare that the research was conducted in the absence of any commercial or financial relationships that could be construed as a potential conflict of interest.

## Publisher’s Note

All claims expressed in this article are solely those of the authors and do not necessarily represent those of their affiliated organizations, or those of the publisher, the editors and the reviewers. Any product that may be evaluated in this article, or claim that may be made by its manufacturer, is not guaranteed or endorsed by the publisher.
