# Supplementary material for: Right Ventricle to Pulmonary Artery Coupling Predicts the Risk Stratification in Patients With Systemic Sclerosis-Associated Pulmonary Arterial Hypertension
Source: Front Cardiovasc Med. 2022 May 11;9:872795. doi: 10.3389/fcvm.2022.872795 (PMC9130575; doi:10.3389/fcvm.2022.872795)
Supplement: Supplementary file 1 [file Table_1.DOCX]

**Supplementary Material. Simplified risk stratification for pulmonary arterial hypertension**

| Prognostic factors | Low-risk | Intermediate-risk | High-risk |
| --- | --- | --- | --- |
| I: WHO-FC | I, II | III | IV |
| II: 6MWD | > 440 m | 165~440 m | < 165 m |
| III: NT-proBNP /BNP or RAP | BNP < 50 ng/L,  NT-proBNP < 300 ng/L or RAP < 8 mmHg | BNP 50~300 ng/L,  NT-proBNP 300~1400 ng/L or  RAP 8~14 mmHg | BNP > 300 ng/L,  NT-proBNP > 1400 ng/L or RAP > 14 mmHg |
| IV: CI or SvO_2_ | CI ≥ 2.5 L/min/m^2^ or SvO_2_ > 65% | CI 2.0~2.4 L/min/m_2_ or  SvO_2_ 60%~65% | CI < 2.0 L/min/m^2^ or  SvO_2_ < 60% |

Abbreviations: WHO-FC, World Health Organization functional classification; 6MWD, 6minute walk distance; NT-proBNP, N-terminal pro B-type natriuretic peptide; BNP, B-type natriuretic peptide; CI, cardiac index; SvO_2_, venous oxygen saturation
